# The Application of Liquid Biopsy for the Development and Validation of a Non-Invasive Screening and Diagnosis Test for Endometrial Premalignant and Malignant Lesions: A Prospective Innovative Pilot Study

**DOI:** 10.3390/cancers17071078

**Published:** 2025-03-23

**Authors:** Giuseppina Esposito, Giuseppe D’Angelo, Luigia De Falco, Eloisa Evangelista, Giovanni Savarese, Antonio Fico, Federica Cinque, Pierluigi Giampaolino, Attilio Di Spiezio Sardo, Giuseppe Bifulco, Luigi Della Corte

**Affiliations:** 1 Department of Public Health, University of Naples Federico II, 80131 Naples, Italy; giusyesposito890@gmail.com (G.E.); cinquefede5@gmail.com (F.C.); pierluigi.giampaolino@unina.it (P.G.); attilio.dispieziosardo@unina.it (A.D.S.S.); giuseppe.bifulco@unina.it (G.B.); 2 AMES, Polidiagnostic Strumental Centre, Srl, 80013 Naples, Italy; defalcol@centroames.it (L.D.F.); eloeva@hotmail.it (E.E.); giovanni.savarese@centroames.it (G.S.); centroames@libero.it (A.F.); 3 Fondazione Genetica per la Vita Onlus, 80132 Naples, Italy; 4 Department of Neuroscience, Reproductive Sciences and Dentistry, School of Medicine, University of Naples Federico II, 80131 Naples, Italy

**Keywords:** endometrial cancer, liquid biopsy, circulating tumor DNA, non-invasive diagnostics, next-generation sequencing, personalized medicine, cancer monitoring

## Abstract

**Background/Objectives**: Endometrial cancer (EC) is a common malignancy in developed countries, with incidence closely linked to lifestyle factors and genetic predispositions, notably Lynch syndrome. Traditional biopsy methods for diagnosis and monitoring are invasive. This study aims to develop and validate a non-invasive diagnostic method for EC using liquid biopsy, specifically examining circulating tumor DNA (ctDNA) for its potential in early detection and disease monitoring. **Methods**: A cohort of 63 patients with EC or atypical endometrial hyperplasia (AEH) was recruited from the Gynecological Unit of the Azienda Ospedaliera Universitaria Federico II. Plasma samples were processed to extract ctDNA, which was sequenced and analyzed for mutations. Matched tumor tissue and germline DNA were also examined to confirm mutation concordance and assess potential genetic predispositions. **Results**: Pathogenic mutations were identified in plasma ctDNA in 59 out of 63 cases (93%), with a 65% concordance between plasma ctDNA mutations and those found in solid tumor samples. Key mutations in genes such as *PTEN*, *PIK3R1*, and *KMT2C* were significantly associated with a higher tumor grade and advanced stage disease, such as myometrial infiltration. **Conclusions**: Liquid biopsy shows promise as a minimally invasive diagnostic and monitoring tool for EC, offering real-time insights into tumor biology. The high mutation concordance between the plasma ctDNA and tumor tissue underscores the potential of a liquid biopsy in managing EC, particularly for patients at risk of recurrence. Further longitudinal studies are needed to establish ctDNA as a standard tool in EC diagnosis and monitoring.

## Simple Summary

Endometrial cancer is a common type of cancer in women, and early detection is essential for effective treatment and improved outcomes. Traditional diagnostic methods often rely on invasive procedures, which can be uncomfortable and risky. Our research explores the use of a non-invasive method called liquid biopsy, which analyzes small fragments of tumor DNA circulating in the blood. By using advanced sequencing technologies, we were able to identify specific genetic mutations linked to endometrial cancer in a large group of patients. This approach could revolutionize how endometrial cancer is diagnosed and monitored, offering a less invasive, more precise, and patient-friendly option. Our findings lay the foundation for further development of liquid biopsy techniques, which may help doctors detect cancer earlier, tailor treatments to individual patients, and track disease progression more effectively. This study represents a step forward in making cancer diagnostics more accessible and personalized.

## 1. Introduction

Endometrial cancer (EC) is the fourth leading cancer in women in developed countries, posing a significant health burden among women [1]. In Europe, the incidence of EC varies widely, with higher rates observed in Northern and Western Europe compared to Southern and Eastern regions. 

According to the European Cancer Information System, approximately 130,000 new cases were diagnosed and over 30,000 deaths occurred due to endometrial cancer in Europe in 2020 [2]. This rising incidence is closely linked to increasing rates of obesity, aging populations, and lifestyle factors. 

Epidemiologically, EC primarily affects postmenopausal women, with a median age at diagnosis of around 65 years. Risk factors include prolonged estrogen exposure, obesity, nulliparity, late menopause, diabetes, and hypertension. Additionally, genetic predispositions such as Lynch syndrome significantly increase the risk of developing endometrial cancer. 

Historically, EC has been classified based on histopathological features into two main types: Type I (endometrioid and estrogen-dependent) and Type II (non-endometrioid and estrogen-independent) [3].

Although most ECs are diagnosed early, mainly due to symptomatic postmenopausal metrorrhagia, with a 5-year survival of 90%, up to 20% of the lesions progress to a high-stage carcinoma. Myometrial infiltration and lymph-vascular space invasion (LVSI) are crucial elements for prognosis in EC patients [4]. The advent of molecular profiling technologies has revolutionized the understanding of endometrial cancer, leading to a paradigm shift in its classification. 

The Cancer Genome Atlas (TCGA) Research Network has provided a comprehensive molecular characterization of endometrial cancer [5], delineating four distinct subgroups based on genomic, transcriptomic, and proteomic data. These subgroups are:

- POLE ultramutated: characterized by a high mutation rate due to alterations in the POLE gene, these tumors have a favorable prognosis.
- Microsatellite instability-high (MSI-H): with high levels of microsatellite instability, often associated with Lynch syndrome, these tumors also tend to have a relatively good prognosis.
- Copy-number low: exhibit low levels of somatic copy-number alterations and are typically endometrioid and have intermediate outcomes.
- Copy-number high (serous-like): characterized by extensive copy-number alterations and TP53 mutations, these tumors are analogous to serous carcinomas and have the worst prognosis among the subgroups.

EC is frequently driven by specific genetic alterations, notably in *PTEN*, *PIK3CA*, *ARID1A*, *KRAS*, and, less commonly, *TP53* as well as mismatch repair (MMR) genes (e.g., *MLH1*, *MSH2*, *MSH6*, *PMS2*) [5,6,7]. Among these, *PTEN* mutations have been reported in up to 55–80% of endometrioid endometrial tumors and their precursor lesions, while *PIK3CA* and *ARID1A* often co-occur in endometrial neoplasms, contributing to tumor progression [8,9]. *KRAS* mutations, though less frequent, may also promote oncogenic pathways in endometrial tissue [5,8]. Importantly, these genetic changes are not confined to EC; the same oncogenic drivers are encountered in multiple cancer types, underscoring shared molecular pathways across diverse malignancies. For example, *PTEN* and *PIK3CA* aberrations have been detected in breast and colorectal cancers, where they are sometimes linked to therapeutic resistance or differential prognosis [10,11]. 

Similarly, *ARID1A* mutations are frequently observed in ovarian clear cell and endometrioid carcinomas, suggesting overlapping tumorigenic mechanisms between certain subtypes of gynecologic tumors [12].

These molecular commonalities have practical implications for both diagnostic and therapeutic strategies. As next-generation sequencing becomes increasingly integrated into routine clinical care, identifying mutations in key oncogenes and tumor suppressors can guide precision treatments and inform prognostic assessments [8]. Moreover, the recognition that *PTEN*, *PIK3CA*, and *ARID1A* mutations are shared by endometrial, ovarian, and colorectal cancers provides a rationale for translational research that may benefit multiple patient populations [13]. 

The FIGO 2023 classification provides several key changes regarding diagnostic accuracy and treatment stratification [14,15,16]. Integration of the TCGA molecular classification with the updated FIGO 2023 staging system represents the most substantial advancement in personalized management of endometrial cancer. By combining molecular profiles with precise anatomic staging, therapeutic approaches should be tailored to the individual patient profile thereby optimizing outcomes. 

Accurate diagnosis of EC is critical for effective management and treatment. The most recent guidelines emphasize a multimodal approach, incorporating clinical evaluation, imaging, histopathology, and molecular testing [15,17]. 

Women aged 45 and above who present with abnormal uterine bleeding should be evaluated for EC. Indeed, 90% of women diagnosed with EC exhibit symptoms [18,19,20]. For postmenopausal women presenting with bleeding, a transvaginal ultrasound (TVS) is utilized as a triage tool to determine endometrial thickness (ET). If the ET exceeds 4 mm, the recommended gold-standard diagnostic procedure for EC is an endometrial biopsy [19,20,21]. However, in perimenopausal or premenopausal women who are symptomatic and have EC risk factors, the National Institute of Clinical Excellence advises performing hysteroscopy and a targeted biopsy first [22], as TVS offers limited diagnostic utility in actively menstruating women [23].

The treatment of EC is tailored according to the stage of the disease, histologic subtype, molecular classification, and patient risk factors. 

According to the latest National Comprehensive Cancer Network (NCCN) guidelines, the principal treatment for endometrioid EC confined to the uterus involves total hysterectomy, a bilateral salpingo-oophorectomy and lymph node staging [24], ideally using a minimally invasive technique whenever possible. Young women who wish to preserve fertility and might qualify for fertility-sparing treatments must be informed that these approaches are not considered the standard of care [17]. Moreover, they should be encouraged to attempt conception as soon as complete remission is attained; once childbearing is finalized, definitive radical surgery should be pursued.

On the other hand, malignancies distinct from pure endometrioid carcinoma—such as serous carcinoma, clear cell carcinoma, undifferentiated carcinoma, choriocarcinoma, and malignant mesenchymal tumors (sarcomas)—are managed as high-grade endometrial cancers, and fertility-sparing surgery is therefore not advised [25].

Nowadays, research efforts are focused on the discovery of new non-invasive methods for the diagnosis and comprehension of the tumor molecular architecture in real time.

Liquid biopsy, which involves the analysis of circulating tumor DNA (ctDNA) and circulating tumor cells (CTCs), could play a crucial role in the development and validation of non-invasive diagnostic tests for EC. By providing real-time insights into the molecular landscape of the tumor, a liquid biopsy can help in monitoring treatment response, detecting minimal residual disease, and identifying potential relapse earlier than conventional imaging techniques [26].

In addition, a liquid biopsy offers several advantages over traditional tissue biopsies, including being minimally invasive, allowing for repeated sampling, and providing a comprehensive overview of tumor heterogeneity. 

The current literature has demonstrated the feasibility of using a liquid biopsy for detecting endometrial cancer-specific mutations, microsatellite instability, and other molecular alterations associated with the disease. For instance, studies have shown that ctDNA can be used to identify mutations in genes such as *PTEN*, *PIK3CA*, and *KRAS*, which are frequently altered in endometrial cancer [27,28,29,30]. 

In this study, we aimed to develop and validate a non-invasive screening and diagnostic test for endometrial cancer utilizing Next Generation Sequencing techniques on ctDNA (Liquid Biopsy).

## 2. Materials and Methods

### 2.1. Patient Recruitment

A total of 71 matched tumor-plasma-buffy coat samples were collected in the Gynecological Unit of DAI Materno-Infantile of the Azienda Ospedaliera Universitaria Federico II in Naples, from patients diagnosed with EC or AEH referred to the AMES laboratory in Naples for ctDNA analysis from January 2021 to September 2023. 

The AMES laboratory is accredited (UNI EN ISO) [31] for genetic testing. The study was conducted according to the guidelines of the Declaration of Helsinki. This study was approved by the Ethics Committee of the University of Naples Federico II (protocol number 211/19). 

Inclusion criteria were women 18–80 years old affected by EC stage I-IV or AEH. Exclusion criteria were: age < 18 years or >80 years, current pregnancy, psychiatric disease and/or incapacity of mind and/or will, neoadjuvant chemotherapy, other oncological diseases. All patients signed the informed consent form, agreeing to participate in the study.

Patients included in the study were chosen based on the availability of sufficient plasma, white blood cell buffy coat, and sufficient formalin-fixed, paraffin-embedded EC for NGS analysis. EC tissue and peripheral blood samples were both obtained at the time of hysterectomy, or at the time of hysteroscopy in case of conservative management. Peripheral blood was drawn into EDTA tubes, and plasma and buffy coat fractions were separated within 12 h of collection and stored at −80 °C. 

Regarding the surgical specimen, it was evaluated at our institution by two independent pathologists. In cases of disagreement, a third pathologist reviewed the cases to reach a consensus. Immunohistochemical analysis was performed using specific diagnostic kits for EC, following standardized protocols. These methods ensured precise histopathological and molecular characterization of EC, consistent with the latest international guidelines [8].

### 2.2. Liquid Biopsy Extraction

To eliminate blood cells, the sample was first centrifuged at 1800× *g* for 10 min at 4 °C. The supernatant was then centrifuged again at 16,000× *g* for 10 min at 4 °C to remove any residual cells. A total of 2 mL of plasma was digested with 100 μL of proteinase K buffer for 10 min at 37 °C, after which the ctDNA was isolated using a QIamp Circulating Nucleic Acid kit, following the manufacturer’s instructions. The extracted ctDNA was subsequently quantified via a Picogreen fluorescence assay, with lambda DNA standards serving as the reference.

### 2.3. Tissue Biopsy Extraction

Genomic DNA was isolated using an MGF03-Genomic DNA FFPE One-Step Kit (MagCore Diatech, Diatech Pharmacogenetics, Jesi, AN, Italy) in accordance with the manufacturer’s protocol. Its quality was then verified in triplicate using an FFPE QC Kit (Illumina, San Diego, CA, USA), also following the provided guidelines. The same MGF03-Genomic DNA FFPE One-Step Kit (MagCore Diatech) was employed to extract genomic DNA from fresh tissue samples.

Subsequently, DNA quantification was performed with a Qubit 3.0 Fluorometer in combination with a Qubit dsDNA HS (High Sensitivity) Assay Kit. Following the manufacturer’s instructions, 100 ng of the extracted DNA was used to prepare libraries for a “custom panel” (Kapa HyperPlus Custom Probes, Roche, Basel, Switzerland), as shown in Table 1.

### 2.4. Libraries Preparation and Sequencing

After the target region had been captured and enriched, the resulting DNA libraries were quantified using the Qubit dsDNA HS Assay Kit fluorescent dye method, ensuring equivalent concentrations for each library. Sequencing was then performed on a NovaSeq 6000 platform (Illumina Inc., San Diego, CA, USA) at a mean depth of at least 200×. The reads were aligned to the GRCh37 human reference genome (http://www.ncbi.nlm.nih.gov/projects/genome/assembly/grc/human/index.shtml, accessed on 11 October 2018) via the Burrows–Wheeler Aligner using default settings. Trimming, base calling, coverage analysis, and variant calling were conducted through an in-house bioinformatics pipeline (bcl to fastq v2.20, Isaac Aligner v4, GATK v4, Samtools v1.9, and Bedtools v2).

Variant Call Format (VCF) analysis was carried out in GenomeUp, retaining variants with a quality score above 15 and focusing on small variant consequences (e.g., stop gains, frameshifts, splice-site changes, in-frame deletions or insertions, loss of the start codon [ATG], missense protein alterations, and incomplete terminal codons). Further filtering excluded variants with a frequency above 0.05 in European populations, based on data from the 1000 Genomes Project (https://www.internationalgenome.org/, accessed on 11 October 2018), gnomAD, the Exome Aggregation Consortium (http://exac.broadinstitute.org/, accessed on 11 October 2018), and the Human Gene Mutation Database (HGMD, http://www.hgmd.cf.ac.uk/ac/ accessed on 11 October 2018). Only variants in coding exons or canonical splice sites were considered, and synonymous variants were removed, leaving only rare, high-quality variants (frequency below 0.1%) in dbSNP138 and in our internal repository of more than 1000 exomes.

The “custom panel” of endometrial tumor-related genes is reported in Table 1. The genetic analysis included the identification of mutations in the genes most frequently involved in EC. The identified genes were grouped into key biological families for a better interpretation of the results. Classification was based on their function in major oncogenic pathways and their clinical relevance. Variants were classified according to American College of Medical Genetics and Genomics (ACMG) guidelines [32].

### 2.5. Statistical Analysis

Statistical analysis was performed using the Statistical Package for Social Science, version 17.0 (SPSS, Chicago, IL, USA). Sample characteristics were reported using standard descriptive statistics, with mean ± standard deviation (min to max) in the case of numerical variables and absolute frequencies and percentages in the case of categorical factors. Numerical variables showing highly skewed distribution were described using a median with an interquartile range (25th–75th percentile). Data distribution for continuous variables was assessed with the Shapiro–Wilk’s test. Statistical significance was set at *p* < 0.05.

Additionally, a 2 × 2 contingency table was constructed to evaluate concordance between ctDNA and tissue mutation status (present vs. absent), and Cohen’s Kappa was calculated to quantify agreement beyond chance. We reported both the Kappa coefficient and its 95% confidence interval.

## 3. Results

### 3.1. Patient Characteristics

A total of 71 patients met the inclusion criteria and were primally enrolled in the study. Eight patients were excluded for lack of inclusion criteria. The histological characteristics of the sample collected are summarized in Table 2.

Among the 63 patients included in the study, 35/63 (55.8%) had a histological diagnosis of type 1 endometrioid EC, 24/63 (39.0%) of AEH, 2/63 (2.6%) of sarcoma and 2/63 (2.6%) of type 2 non-endometrioid and estrogen-independent EC. All patients with endometrial cancer underwent radical surgery, while 13/24 patients with diagnosis of AEH in childbearing age (mean age 30.6 ± 5.4 years old) opted for conservative treatment [33]. Clinical and pathological characteristics of enrolled patients are summarized in Table 2, Table 3 and Table 4.

### 3.2. Genetic Analysis

The mean yield of cfDNA extracted from plasma samples was 18.6 ng/mL (range: 6–30 ng/mL). At least one pathogenic mutation in plasma samples was detected in 59/63 tumors (93%) analyzed (χ^2^ = 28; *p* = 0.01) (Table S1). Overall, 63 patients were enrolled in this study: 35 with EC and 24 with AEH. Notably, ctDNA was detected in 21/24 (88%) AEH cases, compared to 34/35 (97%) cases of EC. Although premalignant lesions are commonly assumed to have lower ctDNA detection rates, our findings demonstrate that AEH can still release detectable levels of tumor-derived DNA.

Among the 11 AEH patients who underwent hysterectomy, final histopathological examination confirmed the AEH diagnosis in all cases. The remaining 13 AEH patients, who opted for a fertility-sparing or conservative approach, underwent serial follow-up endometrial sampling or imaging, which documented lesion regression over time. 

To further characterize the detected mutations, we also evaluated the Variant Allele Frequency (VAF) in ctDNA. The VAF for each mutation ranged from 10% to 51%, demonstrating both the presence and relative abundance of tumor-derived DNA in the plasma. This evaluation helps ensure that the detected variants indeed originate from the tumor rather than representing background germline alterations. 

**Figure 1:**
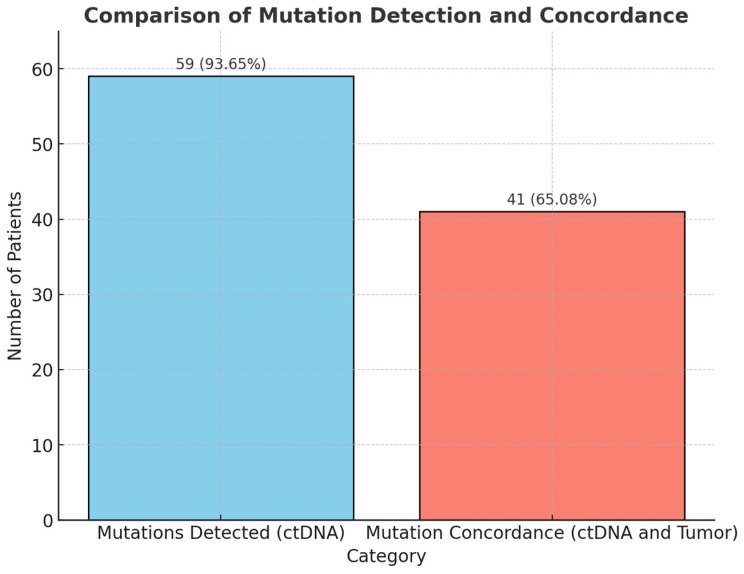
Comparison of mutation detection and concordance between circulating tumor DNA (ctDNA) and tumor tissue in endometrial cancer patients.

The detected mutation in plasma corresponded to the mutation found in the solid tumor in 41 out of 63 cases (65%). As shown in Figure 1, ctDNA mutations were detected in 59 out of 63 cases (93%), and a total of 41 cases (65%) displayed concordance between plasma and tissue.

To further quantify this agreement, we made a 2 × 2 contingency table (Table 5) in order to compare the presence or absence of the relevant mutation in both ctDNA and tumor tissue. Based on these counts, an unweighted Cohen’s Kappa of 0.22 (95% CI: 0.033–0.42) was calculated, indicating a “fair” level of concordance beyond chance.

From these data, 41 cases displayed mutations in both ctDNA and tumor tissue (a = 41), while in 18 cases, mutations were detectable only in ctDNA (b = 18). No cases showed mutations exclusively in the tumor (c = 0), and in four cases, neither ctDNA nor tumor tissue contained the mutation (d = 4). This distribution yielded a Cohen’s Kappa of 0.22 (95% CI: 0.033–0.42), suggesting a fair level of concordance between the two detection methods.

Concordance was higher for AEH (75%) compared to the pooled group of type 1 endometrioid and type 2 non-endometrioid, estrogen-independent EC cases (55%). Among type 2 EC cases, the number of patients was limited (n = 2), so their individual concordance rate was not calculated separately but included within the overall percentage.

The amount of ctDNA in plasma was not related to tumor grading (*p* > 0.05) but was significantly associated with myometrial infiltration (χ^2^ = 25; *p* = 0.001).

Pathogenic mutations identified in plasma or solid tumors were found in the germline in 19% of endometrioid ECs and 14% of AEH cases.

In 42% of endometrioid ECs and 28% of AEH cases, the gene in the germline had a benign mutation that acquired a pathogenic mutation in the plasma or solid tumor. In 38% of endometrioid ECs and 21% of AEH cases, the mutation was not present in the germline (χ^2^ = 26; *p* = 0.03).

**Figure 2:**
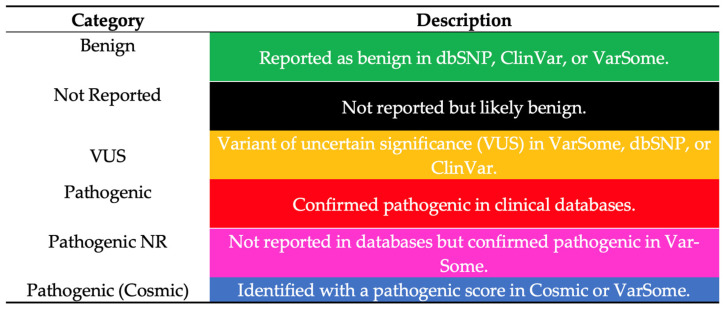
VARSOME and its significance (COSMIC: Catalogue of Somatic Mutations in Cancer). Legend provides a color-coded classification of genetic variants based on their clinical significance. The categories outlined here serve as a reference for interpreting the subsequent VARSOME analysis images.

To further interpret the pathogenic relevance of the mutations identified, we classified each variant using VarSome *(*Figure 2). This tool designates variants as benign, variant of uncertain significance (VUS), or pathogenic, based on established databases (e.g., ClinVar, COSMIC). Notably, a substantial proportion of the variants in our cohort were categorized as pathogenic, underscoring their potential clinical significance.

**Figure 3:**
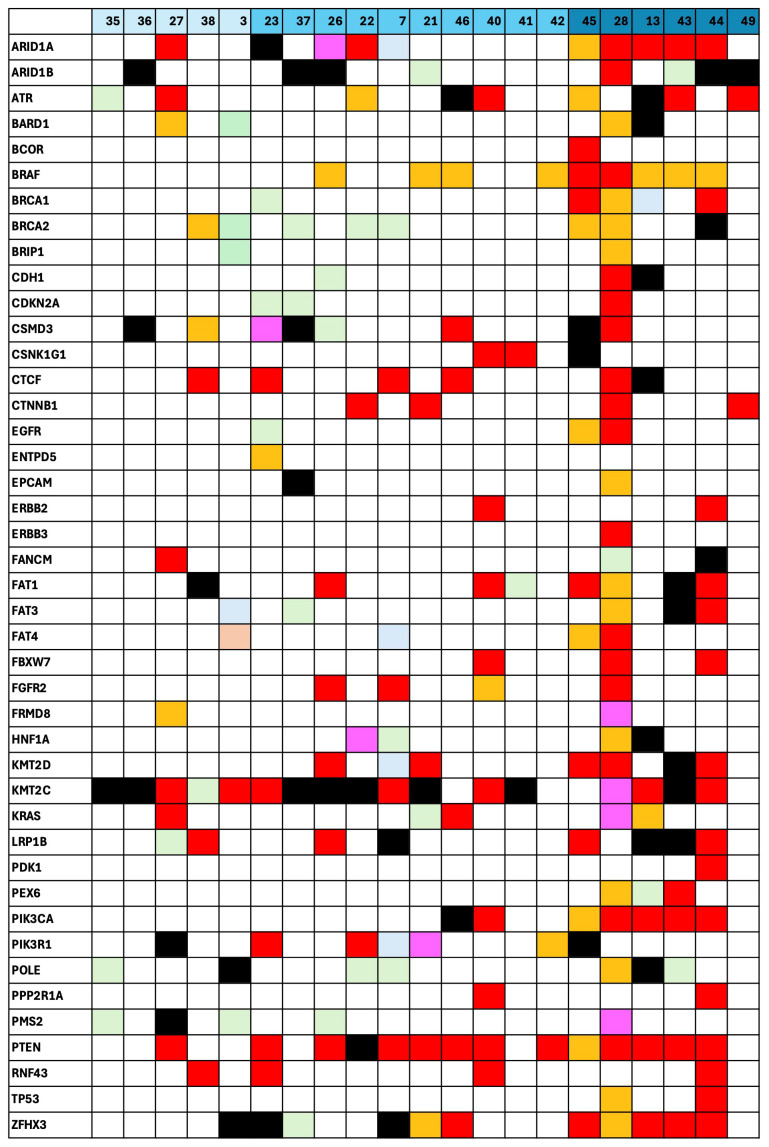
Distribution of mutations (classified by VARSOME) in patients with endometrioid endometrial cancer, stratified by tumor grade. Each bar represents a single patient, with deeper blue shading indicating G3 grading, and each cell within the bar denotes the presence or absence of a key tumor-related mutation (color-coded by VARSOME classification; see Figure 2 for interpretation details).

**Figure 4:**
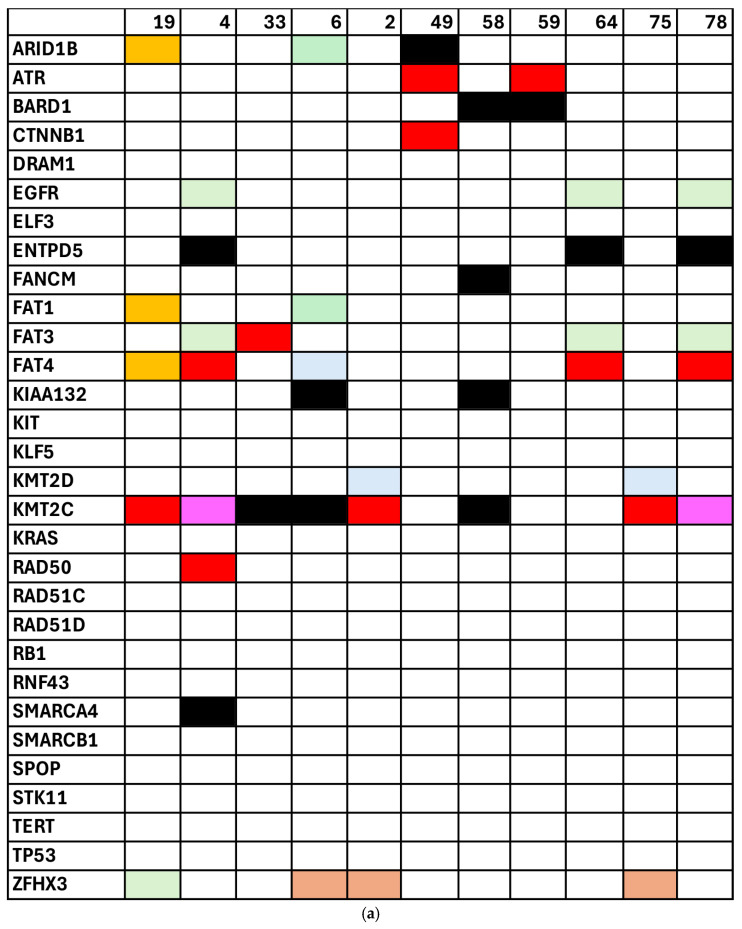

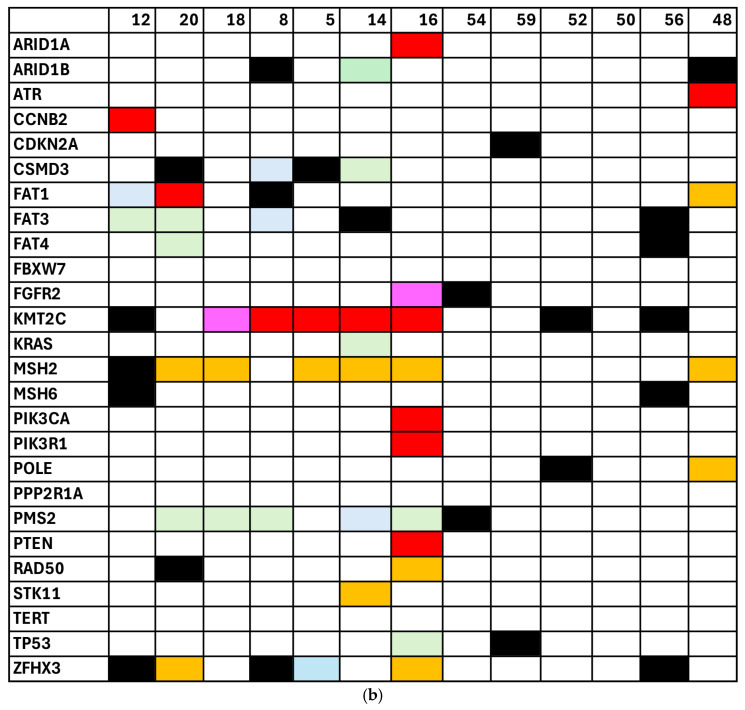
Mutation profiles in patients with atypical endometrial hyperplasia (AEH), categorized by treatment approach. (**a**) Patients who underwent demolitive treatment; (**b**) patients managed conservatively. Each bar represents a single patient and each cell within the bar denotes the presence or absence of a key tumor-related mutation (color-coded by VARSOME classification; see Figure 2 for interpretation details).

We next examined how these mutations correlated with the tumor grade in patients with endometrioid EC, shown in Figure 3. We observed that pathogenic variants in genes such as *PTEN* and *PIK3R1* were more frequently detected in G3 tumors, suggesting a link between these alterations and a more aggressive disease. Finally, Figure 4 displays the mutational landscape in patients diagnosed with AEH, stratified by treatment approach. Figure 4a illustrates the profiles in patients who underwent demolitive (surgical) management, while Figure 4b focuses on those treated conservatively. In both subgroups, certain genes (e.g., *KMT2C*) appeared repeatedly; however, the prevalence and classification of these variants varied between the two treatment groups.

In the study, the genes most frequently found to be mutated were *ATR*, *BRAF*, *CTCF*, *CTNNB1*, *FGFR2*, *KMT2D*, *KMT2C*, *MSH*, *PIK3R1*, *PTEN*, and *ZFHX3*.

The highest concordance in mutations between solid tumors and plasma was observed in *KMT2C*, *PIK3R1*, *PTEN*, and *ZFHX3*. The presence of cfDNA mutations in *PTEN* (χ^2^ = 25; *p* < 0.001) and *PIK3R1* (χ^2^ = 22; *p* < 0.001) in plasma was significantly associated with G3 grading and more than 50% myometrial infiltration.

Similarly, cfDNA mutations in *CTCF* (χ^2^ = 12; *p* < 0.01) and *BRAF* (χ^2^ = 18; *p* < 0.001) were associated with more than 50% myometrial infiltration. Mutations in *ZFHX3*, *P53*, and *PTEN* were predominantly associated with endometrioid EC, while mutations in *KMT2C* were linked to AEH. These findings are summarized in Figure 2, Figure 3 and Figure 4.

## 4. Discussion

In EC, there is a critical need for a non-invasive screening marker to reduce the number of women who undergo invasive diagnostic tests, as well as for a prognostic marker to assist in the timely treatment of endometrial hyperplasia. Such a screening marker could be implemented at the population level, where a positive result would necessitate further diagnostic testing. 

Alternatively, screening could be targeted towards high-risk groups, such as individuals with Lynch syndrome or obesity. These biomarkers must exhibit sufficient sensitivity and specificity, as well as be cost-effective and non-invasive to facilitate widespread use. Diagnostic biomarkers are crucial for confirming the presence of cancer or identifying subtypes, thereby aiding in the management planning based on disease grade and prognosis associated with each biomarker [34]. 

Liquid biopsy has emerged as a groundbreaking advancement in oncology and is seen as a key tool for achieving precision medicine. Beyond blood-based samples, non-invasive sources like saliva, urine, cerebrospinal fluid (CSF), uterine aspirates, pleural effusions, and even stool have also demonstrated promise in detecting tumor-derived elements. These circulating markers include circulating tumor cells (CTCs), ctDNA, circulating tumor microRNA (miRNA), proteins, and exosomes [35].

By offering lower costs and highly reproducible sampling, liquid biopsy may overcome many hurdles posed by traditional, biopsy-based methods for diagnosing endometrial cancer (EC) [26]. The collection of fluid samples—whether from blood, urine, lavage, cerebrospinal and peritoneal fluids, or saliva—is minimally invasive, and these fluids can then be analyzed for ctDNA/cell-free DNA (cfDNA), circulating tumor cells, proteins, and other circulating biomarkers such as RNA, vesicles, and platelets [36,37].

Although this approach has been more thoroughly investigated in other malignancies (breast, colorectal, and prostate cancers), research into a liquid biopsy for EC remains limited, and it has yet to enter widespread clinical application. Nonetheless, the recent adoption of molecular classifications shows that a liquid biopsy could supply valuable diagnostic and prognostic insights, allowing for better screening, monitoring, and patient stratification in EC [28,36,37].

Evidence suggests that elevated quantities of ctDNA/cfDNA in the bloodstream make EC biomarkers particularly easy to detect in more advanced disease stages [28,38]. As an example, Bolivar et al. reported that 94% of a 48-patient cohort carried at least one EC-associated mutation—commonly in CTNNB1, *KRAS*, *PTEN*, or *PIK3CA*—although only one-third of those cases shared an identical mutation in the tumor itself [28]. Meanwhile, a larger study by Danziger et al. examining 193 patients demonstrated that 94% had detectable ctDNA, 48% of whom showed a potentially targetable mutation or biomarker [39]. These findings point to liquid biopsy as a workable solution in EC cases where tumor tissue may be inaccessible, potentially broadening patient eligibility for clinical research.

If diagnosed early, most EC patients benefit from a good prognosis. However, certain individuals still face a recurrence post-surgery and predicting which cases will recur remains a challenge under the current risk classification frameworks. This gap in clinical management likely stems from tumor heterogeneity and early tumor spread [40]. A liquid biopsy counters these issues by enabling an easy and real-time sampling method that can enhance insights into tumor heterogeneity, outperforming traditional, tissue-based biopsies in terms of accessibility and the breadth of information it provides.

In our study, we compared cfDNA mutations found in plasma samples with those identified in solid tumor tissues, and further compared these findings with germline DNA from the same patients to identify de novo mutations. Genetic alterations were detected in 93% of the analyzed samples using targeted sequencing and the detected mutation in plasma corresponded to the mutation found in the solid tumor in 41 out of 63 cases (65%). 

The Cohen’s Kappa statistic of 0.22 suggests that the agreement between ctDNA- based and tissue-based mutation detection exceeds chance alone, yet remains modest. Several factors may influence this level of concordance, including the sensitivity of ctDNA assays, tumor heterogeneity, or the timing of blood sample collection.

Interestingly, our results demonstrate a high ctDNA detection rate across both endometrial carcinoma (97%) and atypical endometrial hyperplasia (88%), supporting the concept that premalignant lesions can already release tumor-derived DNA into circulation. 

However, the even higher rate in invasive EC aligns with prior evidence that more advanced lesions typically shed greater amounts of ctDNA. These findings are consistent with reports in other cancers where ctDNA detection increases with disease progression, yet remains possible at the premalignant stage [41,42,43,44,45]. From a clinical perspective, the substantial ctDNA positivity in AEH (88%) underscores the potential role of a liquid biopsy in identifying or monitoring patients at risk of progression, especially those undergoing fertility-sparing management. 

Nevertheless, our pilot data underscore the need for larger, prospective trials to clarify how a liquid biopsy might reliably capture the tumor mutational landscape in endometrial cancer.

The genes most frequently mutated in our cohort included *ATR*, *BRAF*, *CTCF*, *CTNNB1*, *FGFR2*, *KMT2D*, *KMT2C*, *MSH*, *PIK3R1*, *PTEN*, and *ZFHX3*, consistent with previously described genomic patterns in primary carcinomas [5,46,47,48]. Notably, cfDNA mutations in *PTEN* were frequently associated with *PIK3R1* mutations [5], and in our cohort, they correlated with a higher tumor grade (G3) and more than 50% of myometrial infiltration. Additionally, the rate of ctDNA-positive cases was significantly correlated with myometrial and lymphovascular infiltration, as well as a histological grade, in agreement with recent studies [28].

Moreover, we observed that pathogenic mutations identified in plasma or solid tumors were present in the germline of 19% of endometrioid EC cases and 14% of AEH cases. In 42% of endometrioid EC cases and 28% of AEH cases, the gene carried a benign germline mutation that acquired malignancy in the plasma or solid tumor.

These observations point toward possible genetic predisposition patterns to EC. However, long-term monitoring of the study cohort will be crucial to validate the clinical utility of measuring ctDNA for enhancing risk classification in EC patients.

In this regard, Pereira et al., by analyzing a retrospective cohort of gynecologic tumors (ovary and endometrial tumors), found lower survival rates in patients with detectable ctDNA levels at surgery [30].

Liquid biopsy has frequently been advocated as a relatively non-invasive tool for early cancer detection or screening in individuals at heightened risk. However, its broader application in clinical settings still faces certain limitations. Somatic mutations in key driver genes are not confined solely to malignant processes. For example, one recent study found that 79% (19 out of 24) of women with endometriosis exhibited mutations in genes such as *ARID1A*, *KRAS*, and *PIK3CA* within the epithelial cells of the endometriotic lesions analyzed [49].

A more conservative yet valuable clinical application for a liquid biopsy in EC could be utilizing it as a non-invasive method to monitor patients for recurrence following hysterectomy or for those who underwent conservative management of atypical hyperplasia.

In this setting, an initial characterization of the primary tumor’s molecular alterations is crucial, since any mutations found in the tumor could then be monitored over time through blood samples. Based on the current study—where a next-generation sequencing (NGS) panel focused on just four genes—over 90% of individuals with EEC displayed at least one detectable mutation, indicating that these patients could be followed with serial liquid biopsies. However, it should be established whether such plasma-based mutations can remain traceable years after hysterectomy and whether their detection might function as an early warning of cancer recurrence.

To our knowledge, this is the first study reporting on such a large cohort of patients. The strength of our study rests on the feasibility of using cfDNA as a biomarker for endometrial cancer. A significant portion of patients exhibited detectable mutations in plasma that corresponded with those found in solid tumor samples. The high concordance, especially for genes such as *PTEN*, *PIK3R1*, and *KMT2C*, underscores the potential clinical utility of a liquid biopsy.

However, our study has limitations, including the absence of follow-up blood samples, which are necessary to validate these findings, determine the clearance kinetics of cfDNA mutations post-surgery, and assess the predictive value of cfDNA reappearance for tumor recurrence. A more formal prospective study involving multiple blood samples would be required to establish the timeline for mutation clearance in plasma following hysterectomy. Additionally, prospective studies could help to determine if early-stage patients with plasma-based mutations detected at the time of surgery are at an increased risk of recurrence.

## 5. Conclusions

In conclusion, our findings indicate that a liquid biopsy, utilizing NGS to analyze cfDNA, holds promise for both the early detection and ongoing monitoring of endometrial cancer. Notably, ctDNA analysis showed a high overall detection rate (93%) among patients with endometrial pathology, with particularly high positivity in EC (97%) but also a substantial rate (88%) in AEH. 

Overall, these data suggest that molecular alterations can appear early in the neoplastic process and that non-invasive lesions such as AEH may already harbor tumor-associated mutations detectable in plasma.

The detection of specific mutations in genes such as *PTEN*, *PIK3R1*, *CTCF*, and *BRAF*, which correlate with advanced disease features like high-grade tumors and deep myometrial infiltration, suggests that cfDNA analysis could become a crucial tool in the clinical management of endometrial cancer. Additionally, the identification of mutations present in both germline and acquired tumors underscores the importance of genetic predisposition and the need for further research to understand these patterns.

Future studies should aim to improve cfDNA extraction and sequencing methods to enhance sensitivity and specificity, while also establishing standardized protocols for clinical use. Longitudinal studies will be essential to track the post-operative clearance of cfDNA mutations and to evaluate the potential of cfDNA as a predictive marker for recurrence. Moreover, prospective, multi-institutional trials with extended patient follow-up are warranted to validate the clinical utility of ctDNA-based monitoring. Such investigations should correlate fluctuations in ctDNA levels with imaging findings and clinical outcomes, defining robust cutoff values for ctDNA positivity. Ultimately, demonstrating improved disease surveillance or earlier detection of relapse through ctDNA would further support the integration of a liquid biopsy into personalized treatment approaches.

By integrating a liquid biopsy into the diagnostic and monitoring framework, it may be possible to improve personalized treatment strategies and overall outcomes for patients with endometrial cancer.

## Figures and Tables

**Table 1:** Comprehensive classification of genes associated with endometrial cancer, grouped into biologically relevant families. The table includes the gene name or family, its biological function, and its clinical significance in the context of tumor progression and potential therapeutic implications.

Gene Name/Family	Genes	Function	Clinical Significance
PIK3 Pathway	*PIK3CA*, *PIK3R1*, *PDK1*, *AKT1*, *PPP2R1A*	PI3K pathway regulation, cell growth and metabolism	Frequent mutations in endometrial cancer, affecting tumor progression
MMR Genes	*MLH1*, *MSH2*, *MSH6*, *PMS2*, *EPCAM*	Mismatch repair, maintaining genomic stability	Defects lead to microsatellite instability and high mutation burden
Wnt Pathway	*CTNNB1*, *APC*, *CSNK1G1*	Wnt signaling, cell adhesion and differentiation	Wnt pathway mutations contribute to tumor development
Hippo Pathway	*FAT1*, *FAT3*, *FAT4*	Hippo signaling, cell proliferation control	Hippo pathway mutations linked to aggressive tumors
Notch Pathway	*NOTCH1*, *NOTCH2*, *NOTCH3*	Notch pathway, cell fate determination	Notch pathway alterations affect cell communication and growth
TGF-Beta Pathway	*SMAD2*, *SMAD3*, *SMAD4*	TGF-beta signaling, cellular differentiation and apoptosis	TGF-beta pathway disruption leads to tumor progression
DNA Damage Repair	*BRCA1*, *BRCA2*, *PALB2*, *RAD50*, *RAD51C*, *RAD51D*, *BARD1*, *NBN*, *MRE11*	DNA damage response and repair	Defects increase susceptibility to cancer and impact therapy response
Chromatin Remodeling	*ARID1A*, *ARID1B*, *SMARCA4*, *KMT2C*, *KMT2D*, *MED12*, *CTCF*	Chromatin remodeling and transcription regulation	Alterations affect chromatin accessibility and gene regulation
Growth Factor Signaling	*FGFR2*, *PDGFRA*, *ERBB2*, *ERBB3*, *KIT*, *MET*	Growth factor receptor signaling and oncogenic activation	Oncogenic activation promotes tumor aggressiveness
Tumor Suppressors	*TP53*, *PTEN*, *RB1*, *CDKN2A*, *ZFHX3*	Tumor suppression and cell cycle control	Loss of function leads to uncontrolled cell proliferation
Other Oncogenes	*KRAS*, *NRAS*, *BRAF*, *MAPK1*, *MAP2K6*, *SPOP*, *FBXW7*, *STK11*	Oncogenic signaling and proliferation	Key oncogenic drivers affecting therapy response
Additional Genes	*BCL2*, *BCOR*, *ERBB3*, *LIN54*, *RNF43*, *HRAS*, *SMARCB1*, *HNF1A*, *FOXL2*, *TERT*, *CDH1*, *IMP3*, *FRMD8*, *CCNB2*, *ELF3*, *ENTPDS*, *KLF5*, *COROZA*, *PEX6*, *LAMB1*, *BRIP1*, *FANCM*, *STK11*, *MAPK1*, *SPOP*, *RB1*, *SMARCA4*, *KIAA1324*	Pathway-specific function	Mutation-driven alterations in endometrial cancer

**Table 2:** Clinical and pathological characteristics of enrolled patients.

Histology	Number of Cases	Percentage (%)	Age, Mean	Dev. Std.
Endometrioid EC *	35	55.8	61.8	10.6
Atypical Endometrial Hyperplasia (AEH)	24	39.0	47.4	12.9
Sarcoma	2	2.6	44.5	3.5
Type 2 non-endometrioid and estrogen-independent EC *	2	2.6	74.04	4.2
Total	63	100	56.0	11.1

* EC = Endometrial Cancer.

**Table 3:** Atypical hyperplasia and its treatment.

Treatment	N	Mean Age	Dev. Std.	SEM *
Conservative	13	30.6	5.4	2.4
Demolitive	11	56.4	9.5	1.8

* SEM = Standard Error of the Mean.

**Table 4:** Histological characteristics of endometrioid endometrial cancer and surgery.

Endometrioid Endometrial Cancer			
Grading	G1 32% (11/35)	G2 39% (14/35)	G3 28% (10/35)
Miometrial invasion	<50% 65% (23/35)	>50% 35% (12/35)	
Limphovascular invasion	No 64% (22/35)	Yes 36% (13/35)	
Type of surgery	Laparoscopy 15% (5/35)	Laparotomy 25% (9/35)	Robotic assisted Laparoscopy 60% (21/35)

**Table 5:** Two-by-two contingency table summarizing the presence (+) or absence (−) of the relevant mutation in circulating tumor DNA (ctDNA) versus tumor tissue (N = 63): ‘a’ indicates cases positive for both ctDNA and tissue, ‘b’ positive only in ctDNA, ‘c’ positive only in tissue, and ‘d’ negative in both.

	Tumor Tissue: Mutation+	Tumor Tissue: Mutation–
ctDNA: Mutation+	41 (a)	18 (b)
ctDNA: Mutation−	0 (c)	4 (d)

## Data Availability

The raw data supporting the conclusions of this article will be made available by the corresponding authors on request.

## Supplementary Materials

The following supporting information can be downloaded at: https://www.mdpi.com/article/10.3390/cancers17071078/s1, Table S1: Comprehensive List of the Analyzed Mutations.
